# Crosstalk between Substrates and Rho-Associated Kinase Inhibitors in Cryopreservation of Tissue-Engineered Constructs

**DOI:** 10.1155/2017/1380304

**Published:** 2017-10-19

**Authors:** Arindam Bit, Awanish Kumar, Abhishek Kumar Singh, Albert A. Rizvanov, Andrey P. Kiassov, Pradeep Kumar Patra, Munish Kumar, Akalabya Bissoyi

**Affiliations:** ^1^Department of Biomedical Engineering, National Institute of Technology, Raipur 492010, India; ^2^Department of Biotechnology, National Institute of Technology, Raipur 492010, India; ^3^Department of Biochemistry, University of Allahabad, Allahabad 211002, India; ^4^Institute of Fundamental Medicine and Biology, Kazan Federal University, Kazan 420008, Russia; ^5^Department of Biochemistry, Pt. JNM Medical College, Raipur 492001, India

## Abstract

It is documented that human mesenchymal stem cells (hMSCs) can be differentiated into various types of cells to present a tool for tissue engineering and regenerative medicine. Thus, the preservation of stem cells is a crucial factor for their effective long-term storage that further facilitates their continuous supply and transportation for application in regenerative medicine. Cryopreservation is the most important, practicable, and the only established mechanism for long-term preservation of cells, tissues, and organs, and engineered tissues; thus, it is the key step for the improvement of tissue engineering. A significant portion of MSCs loses cellular viability while freeze-thawing, which represents an important technical limitation to achieving sufficient viable cell numbers for maximum efficacy. Several natural and synthetic materials are extensively used as substrates for tissue engineering constructs and cryopreservation because they promote cell attachment and proliferation. Rho-associated kinase (ROCK) inhibitors can improve the physiological function and postthaw viability of cryopreserved MSCs. This review proposes a crosstalk between substrate topology and interaction of cells with ROCK inhibitors. It is shown that incorporation of ionic nanoparticles in the presence of an external electrical field improves the generation of ROCK inhibitors to safeguard cellular viability for the enhanced cryopreservation of engineered tissues.

## 1. Introduction

Tissue engineering originates from reconstructive surgery where direct transplantation of donor tissue is practiced to repair the function of damaged tissue [1]. Many difficulties arise with direct transplantation due to insufficient donor organs, rejection of the donor organ, and risk of pathogen transmission. An autologous tissue engineering transplant can therefore be an excellent alternative to direct transplantation of donor organs. Molecular self-assembly in biological systems provides the basis of a wide variety of complex structures. These include the class of mechanically superior biological structures based on microstructural features occurring naturally. These in turn serve as a good platform for the growth of cells and tissues. The biomaterials require some significant properties depending on the functionality of the final tissue-engineered construct. Various properties of the biomaterials such as biocompatibility, shape, size, porosity, and thermal and mechanical characteristics must be optimized prior to engineering the required tissue [2–4]. Biocompatibility is the ability of a material to perform with an appropriate host response in a specific situation [5]. All biomaterials may not have the same degree of biocompatibility. In many cases, surface properties have to be altered in order to enhance the interaction of such substrates with the host or biological fluid and suppress adverse immune response [6, 7]. The surface properties of the bioengineered constructs can be altered by various physical modalities such as UV irradiation, plasma etching, and corona discharge. Various chemical methods have also been used such as covalent attachment, chemical grafting, photografting, plasma polymerization, grafting with ionization radiation, and self-assembled monolayer formation to improve the host response and the biocompatibility of the materials.

Degradability is also a major parameter that must be considered for materials that are used as implants in tissue regeneration. For instance, it might result in excessive drug release and possible severe side effects. Moreover, diffusion may cause swelling and leaching of the implants. During swelling, ions or fluid from the tissue is transported into the biomaterial resulting in reduced elasticity of the substrate causing static fatigue or crazing. Similarly, leaching is the process in which one component of the biomaterial dissolves into the surrounding fluid phase and can cause local biological reactions to the released products, reducing both fracture strength and Young's modulus of the material [7]. Furthermore, dissolution of polymers and ceramics is a more frequent phenomenon. Depending on the nature of the polymer, ceramics and calcium-based materials such as calcium hydroxyapatite, calcium phosphates, and bioactive glasses [8] also tend to undergo dissolution. In spite of the advantages that are apparent in the field of tissue engineering, one of the major drawbacks is the long-term storage and transportation of devices for transplantation procedure. Thus, cryopreservation is the method of choice to address the issues of long-term storage and transportation with maximum efficacy of storage procedure. It is anticipated that a broad range of tissue-engineered products such as ready-made and patient-specific devices as well as new cell-based services will be in high demand. The potential future application of tissue engineering is however dependent upon the timely provision of the regenerative medicinal products to patients [9, 10]. Thus, the use of biomaterial-based tissue constructs as regenerative medicines is important because they can be preserved at low temperatures. An integral approach to the clinical application should involve the evaluation of cryopreservation performance for practical production, that is, the manufacturing process must accommodate the presence of large number of cells in the matrix or scaffold.

Using cryopreservation, priority is given to maintaining the integrity of the membranous structure of cell sheets, tissues, and laboratory-produced organs in a bioethical manner. Several strategies for cryopreservation include ultra-rapid freezing and thawing, controlled-rate freezes, freezing with nonpenetrating polymers, vitrification, and equilibrium freezing. Moreover, there are several important factors for successful cryopreservation including composition of the cryopreservation medium and nature of the cryoprotectants, the freezing-thawing procedure, and the intrinsic susceptibility of the cells to freeze damage. We will discuss different types of substrates used in tissue-engineered constructs and their effects on the functionality of tissues following cryopreservation.

## 2. Substrates for Tissue Engineering Constructs

A scaffold is a three-dimensional (3-D) construct which serves as a temporary support for isolated cells to grow and differentiate into a new tissue that can be transplanted into the organism. To a great extent, the design of the scaffold determines the functional performance of the construct. The optimum characteristics of a scaffold depend on its porosity to allow significant transport of nutrients toward the cells, while also allowing the removal of metabolites, and the optimum in growth of cells into the matrix. Additionally, the scaffold should have suitable mechanical properties comparable to *in vivo* tissue at the site of implantation and should be easily connected to the host vascular system [11]. Furthermore, the scaffold material should also be biocompatible and degrade with tissue regeneration and remodeling of the extracellular matrix (ECM). The surface should promote cell attachment and proliferation. Several natural and synthetic materials are widely used as substrates for tissue engineering constructs [12, 13]. In soft tissue applications such as skeletal muscle or cardiovascular substitutes, polymers are mainly used, whereas scaffolds for hard tissue engineering applications such as bone substitutes are fabricated from a wider variety of classes of materials, namely, polymers, ceramics, composites, or metals. Copolymers of two or more polymers are also commonly used [14]. Additionally, reinforcement molecules are frequently added to increase the overall mechanical properties of the substrates. Various materials used for scaffold construction are listed in Table 1 [15–20].

The design of a scaffold ultimately determines the functionality of the grown tissue [21, 22], taking into account the material, its fabrication, and appearance, that is, shape, size, and micropattern. The different types of scaffolds include membranes, cell sheets, microencapsulation, and microcarrier beads. The membrane-based scaffolds have ideal characteristics [23, 24] including optimal biocompatibility, good porosity to provide high hydraulic permeability, a distinct molecular weight cut-off, surface morphology to reduce interaction with body fluids, sufficient chemical, mechanical and thermal stability to withstand the pressure and the sterilization process, and a large surface area relative to volume that allows construction of small integrated devices with high operational capacities. In contrast, biocompatibility, biodegradability, and flexibility are the ideal characteristics of microcarrier beads. Chemical and mechanical stability should be provided to encapsulate cells for their optimal immobilization. At the same time, cellular sheet integrity should be maintained. However, storage of the encapsulated cells within construct requires mechanism of cryopreservation. Thus, in process of ensuring cellular viability during cryopreservation, physic-chemical responses of substrates play a significant role for enriching construct with enriched microenvironment.

## 3. The Role of Substrates in Cryopreservation of Constructs

Cryopreservation of alginate fibrin beads along with bone marrow-derived mesenchymal stromal cells has already been studied [10, 25], allowing a significant rate of cell proliferation and greater cell viability. The integrity of alginate beads was found to be maintained as alginate hydrogel has high water content. Moreover, no significant difference was observed in the microstructure of the bead after cryopreservation. However, a slight crumpling of the beads was observed due to dehydration that ultimately did not hamper the cell physiology. The extracellular environments also remained unaltered. Thus, alginate beads were found to be highly biocompatible for cell encapsulation and could cater to immediate patient needs and aid in the differentiation of bone marrow cells.

The vitrification method for chondrite sheets using different cryoprotectant solutions was studied by Maehara et al. [26]. The minimum volume cooling method, coating of the sheets with poly-L-lysine (PLL), and exposure to liquid nitrogen vapors were found to be the most efficient methods for its cryopreservation [27]. The microstructure of the sheets is not affected even if the sheet breaks. The maintenance of membranous structure was found to be important for the overall functionality of the cells.  Table 2 summarizes the effects of all substrate properties on the outcomes of cryopreservation.

Li et al. [9] carried out *in vitro* and *in vivo* studies using cryopreserved alginate-poly-L-lysine-alginate (APA) microcapsules containing erythropoietin- (Epo-) secreting myoblasts. The experimental outcomes show that higher cell load resulted in a lower Epo reduction. This indicates that a lower implant dose is sufficient to produce a therapeutic effect *in vivo*. Higher cryoprotectant concentrations turned out to have more favorable effects on microcapsule morphology. However, a major drawback of using higher concentrations of cryoprotectant is its effect on cell viability. Overall morphology was also assessed and cell size remained stable. These results confirm the high chemical resistance of the microcapsule. A significantly higher hematocrit level was observed in all the animals implanted with microcapsules when compared with Hank's balanced salt solution (HBSS) control group. Animals implanted with the frozen microencapsulated cells stored using 10% DMSO cryoprotectant solution showed higher hematocrit levels. The angiogenic effects of Epo might be responsible for the presence of several blood capillaries surrounding the cell-loaded microcapsule clump. This neovascularization may suggest that the angiogenic molecule could play a major role in the long-term functionality of this type of cell-loaded system. The benefits of preserving the microcapsules for a longer period of time were evaluated and no significant differences were found, thereby confirming the safety of employing cryopreserved microcapsules. Despite the promising results obtained in this study, the reduction in Epo release after cryopreservation of microcapsules should be minimized for future improvement of cryopreservation protocols [28].

Hang et al. [29] compared the vitrification and freezing of microencapsulated islets in agarose beads. Crank's equation was used to determine the diffusion coefficient values of solutes in the beads. The diffusion coefficient varied with their increase in molecular weight after cryopreservation as compared to unpreserved normal agarose beads. Moreover, variation in effective diffusion coefficients produced an alteration in the structure of the molecular network within the agarose hydrogel after cryopreservation. In the case of substances with lower molecular weights, such as urea and glucose, and higher molecular weight such as bovine serum albumin, the diffusion coefficients were practically unchanged in agarose beads before and after the cryopreservation process. However, the diffusion coefficients for intermediate molecular weight compounds such as vitamin B12, insulin, and lysozyme exhibited a significant change in their values in the case of agarose beads after cryopreservation. Deformation and failure behaviors of the agarose hydrogel before and after cryopreservation were performed using a compression testing machine. After freezing and thawing, the agarose gel membrane showed cracks and about 4% of the islet cells were either partially or totally naked. In comparison, the agarose membrane was found intact in vitrified encapsulated islet cells. It was also shown that cryopreservation causes the agarose beads to form crystals and they exhibited several cracks and general damage, whereas agarose beads exhibited normal morphology by vitrification using VS55.

The mechanical properties of 5% agarose hydrogel were examined after cryopreservation and thawing processes. It was noticed that failure stress, failure strain, and Young's modulus all decreased for agarose after freezing and vitrification in comparison to noncryopreserved agarose. It was also seen that the mechanical properties of vitrified agarose beads were comparable to the normal agarose beads.

In a bioartificial pancreas [30], a proper molecular weight cut-off protected encapsulated islets against immune rejection. There was also a rise in insulin secretion with respect to the glucose concentration in the case of vitrified microencapsulated islets compared to frozen encapsulated islets. In a study conducted by Lai et al. [31], it was suggested that cryopreservation processing detaches osteoblasts from the hydroxyapatite (HA) scaffold and the cells suffer significantly higher damage than those cryopreserved in liquid suspension. Thus, some aspect of cell surface interaction is detrimental and involves thermal modulations when osteoblasts grown over HA scaffolds are exposed to cryoprotective agents for two-step freezing. Without temperature change, however, few morphological changes were observed. Enhanced postthaw attachment and viability were achieved for higher densities of osteoblasts grown over the HA scaffold surface, indicating that some aspect of cell-cell interaction is also beneficial. The HA scaffolds with higher porosity were better for cell attachment; however, porosity was not an essential factor for postthaw viability. Other than the desired substrates used in cryopreservation, Rho-associated kinase also acts as a regulatory mechano-transistor to control cellular viability. They stimulate ligand-kinase activator, thereby initiating chain of mechano-sensing cascades in response to cryoprotectant during cryopreservation process.

## 4. Rho-Associated Kinase (ROCK) in Cryopreservation

**Figure 1:**
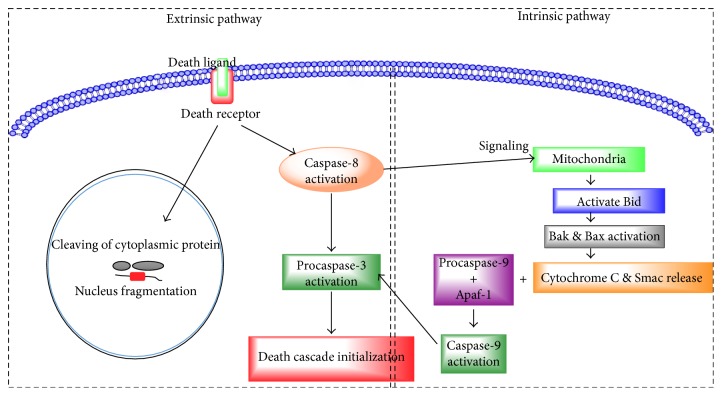
Two distinctive apoptosis pathways. *Extrinsic pathway* death receptors present on the cell membrane ligate due to the binding of their ligands, resulting in the recruitment of Fas-associated protein, activating caspase-8. The active caspase-8 then activates downstream caspases-3 and Bid. Truncated Bid (tBid) activates proapoptotic proteins Bax and Bak on mitochondria. *Intrinsic pathway* triggers permeabilization of the mitochondrial membrane, which further triggers release of cytochrome c, which binds to Apaf-1, which in turn self-associates and binds procaspase-9, resulting in an apoptosome. Transactivation of the complex procaspase-9 to active caspase-9 follows, and the caspase then cleaves and activates downstream caspases.

Apoptotic cell death is dependent on energy and results in shortening of chromatin, cell shrinkage, and fragmentation. Apoptotic death is controlled by two different intrinsic and extrinsic pathways [32, 33]. In the extrinsic pathway, ligands Fas L and tumor necrosis factor-alpha (TNF*α*) bind to death receptors on the cell membrane and activate caspase-8. Caspase-8 further triggers procaspase-3 which ultimately initiates the death cascade by cleaving nuclear DNA and cytoplasmic proteins. Caspase-8 is also reported to be involved in the intrinsic pathway by triggering a signaling cascade through mitochondria for activating Bid that in turn activates Bak and Bax. In this pathway, many stress stimuli such as DNA damage, oxidants, pressure overload, and toxin-induced hypoxia activate proteins such as Bad, Nix, Bim, and Bid (as shown in Figure 1). These proteins activate Bak and Bax, releasing cytochrome c (Cyt c), Smac, and HtrA2 from mitochondria [34–37]. The Cyt c forms a complex with procaspase-9 and Apaf-1 and stimulates the formation of apoptosome that further activate caspase-9. Caspase-9 activates procaspase-3 to initiate the death cascade. Rho-associated kinase (ROCK) is a small Rho-binding protein that belongs to the Rho family with protein kinase activity of threonine/serine along with a molecular mass of nearly 160 kDa [38–41], and a pleckstrin homology (PH) domain rich in cysteine at the carboxyl terminal of the protein. The ROCK family contains two ROCK isoforms, ROCK 1 or p160 ROCK, and ROCK 2. ROCK 1 and ROCK 2 share 65% and 92% of amino acid sequence homology and kinase domain, respectively.

Autoinhibition is also a unique activity of ROCK protein. The carboxyl-terminal region of ROCK acts as an autoregulatory inhibitor of the amino terminal of the kinase domain. In an inactive state, the Rho-binding domain (RBD) of ROCK and the carboxyl-terminal PH domain interact with amino-terminal kinase domains thereby forming a closed autoinhibitory loop. The dynamic form of ROCK is obtained by crosstalk of active GTP bound form of Rho with ROCKRBD that results in suppression of carboxyl-terminal RBD-PH domain by the kinase domain, which leads to an active open kinase domain of ROCK. In addition, caspase-3 is responsible for cleaving the C terminus end of ROCK by which loses its autoinhibitory properties and becomes hyperactive. This hyperactive form of ROCK is a major cause of apoptosis as it acts on various substrates resulting in activation of apoptosis [42]. Terminal domains of both classes of ROCK act differently for generating apoptotic signaling mechanism for cells.

## 5. Targets of ROCK in Apoptotic Signaling

The amino-terminal domains of ROCK 1 and ROCK 2 contain the consensus sequence for phosphorylation of protein targets by serine/threonine protein kinases. The consensus sequence for phosphorylation is R/K-X-X-S/T (R-arginine, K-lysine, S-serine, T-threonine, and X-any amino acid). Moreover, most ROCK marks are cellular macromolecules involved in regulatory actin cytoskeleton. The most common targets of ROCKs are myosin light chain (MLC), and myosin-binding subunit of MLC phosphatase (MYPT1), LIM kinase-1, and kinase-2 at preserved threonines within the activation loop leading to phosphorylation of cofilin proteins, altering the G-actin and F-actin equilibrium. ROCK acts in two ways—either by increasing MLC phosphorylation by directly acting on MLC or by an indirect inhibition of MLC phosphatase activity. The increase of MLC phosphorylation results in actomyosin contraction, whereas inhibition of MLC phosphatase results in smooth muscle contraction [43].

The activation of contractile actomyosin controls the morphological apoptotic events which include blebbing of the plasma membrane, disintegration of the nucleus, and fragmentation.

**Figure 2:**
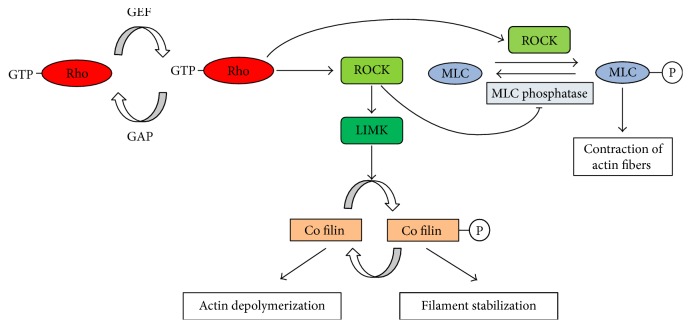
Role of ROCK in the regulation of cytoskeletal proteins.

The phosphatase and tensin homologue (PTEN) is another target for ROCK. The phosphorylation of PTEN using ROCK triggers the phosphatase activity of PTEN, which results in reduction in phosphorylation of Akt, which is crucial for cell survival. This negative regulation of Akt pathway leads to the onset of apoptosis. Another target of ROCK is increased phosphorylation of ezrin, which leads to clustering of Fas and expression of membrane protein resulting in an activation of extrinsic pathway that further initiate apoptosis (as shown in Figure 2) [44]. It leads to investigating another class of substrates to inhibit the apoptotic role of ROCK during the process of cryopreservation.

## 6. ROCK Inhibitors in Cryopreservation

Cryopreservation is a technique which is being used since the 1970s, and its applications have grown immensely [9]. This technique can be employed for the preservation of different cell types, tissues, or even organs at very low temperatures which may fall even below −140°C. However, the process is not that simple and the success rate is very low in postthaw culturing due to the severe toxic conditions and physical stresses created by such low temperatures. At these temperatures, there is natural tendency for intracellular ice formation as well as harmful changes to the chemiosmotic balance of the cell membrane [9]. To overcome this situation, a wide variety of cryoprotective agents such as DMSO and glycerol has been used which certainly increased the survival rate but not to appreciable limits. Thus, there was a real need to look for other protective agents, accelerated by a study conducted by Baust et al. [45]. This group found a marked increase in a cell survival rate when an inhibitor of caspase-3 was added to DMSO [45]. This paper changed the focus of the researchers from studying simple cryoprotective agents to the mechanisms which result in cell death. This has led to much advancement in increasing the survival rate of cells and cultures at the postthaw stage. One such advancement was the use of ROCK inhibitors. Recently, many inhibitors have been used to inhibit the activity of ROCK cleaved by caspase but only two are in wide use. Y-27632 and Fasudil have showed a marked improvement in postthawing viability of the cells. These are compounds which target the ATP-dependent kinase site of activated ROCK without which, it cannot phosphorylate MLC, thus reducing membrane blebbing [44]. Since membrane blebbing is an essential condition for cell apoptosis and is the result of phosphorylation of different substrates such as LIM kinase and MLC, it was observed that Y-27632 efficiently impairs the phosphorylation and thus prevents apoptosis. Another novel ROCK inhibitor is Y-39983 which is considered more effective than Y-27632 [46]. Fasudil is a selective RhoA/Rho kinase (ROCK) inhibitor, which couples vascular remodeling and vasoconstriction in pathogenesis of pulmonary hypertension [47]. The act of ROCK inhibitors in combination with dynamic (or functional) modeling of physic-chemical state of substrates can be proposed for improving outcomes of cryopreservation.

## 7. Crosstalk between Substrate Morphology and ROCK for Improved Cryopreservation Outcome

During cryopreservation, anhydrous cell membranes act as phase-separated membranes under weak tension. The total free energy of the cell membrane (*F*) regulates formation of ROCK under increased concentration of cryoprotectant in extracellular fluid. *F* can be represented as a cumulative contribution of bending elasticity (*F*_b_), membrane surface tension (*F*_*λ*_), and line tension between phases (*F*_*α*_).

(1) $$\begin{array}{c} F=F_{b}+F_{\lambda }+F_{\alpha }. \end{array}$$

Increasing bending elasticity of the cell membrane enhances the ability of the cell to withstand greater stress due to increased concentration of cryoprotectant. At the same time, membrane surface tension needs to be controlled as it regulates the initiation of ROCK synthesis process.

Bending elasticity, membrane surface tension, and line tension between phases can be represented by (2), (3), and (4), respectively [43, 44].

(2) $$\begin{array}{c} F_{b}=\sum_{i=A,B}\iint_{S_{i}}\frac{k_{i}^{c}}{2}{\left(2H-c_{0}\right)}^{2}dS, \end{array}$$

(3) $$\begin{array}{c} F_{\lambda }=\sum_{i=A,B}\lambda \iint_{S_{i}}dS, \end{array}$$

(4) $$\begin{array}{c} F_{\sigma }=\sigma \int_{\partial S}\partial l, \end{array}$$

where *i* = 1, 2 represents the domains of the membrane occupied by incompatible amphiphiles “1” and “2.” *k*_*i*_^*c*^ is represents bending rigidity. *H* and *c*_0_ are the mean and spontaneous membrane curvature. *λ* and *σ* are the surface and line tension [48].

On the other hand, increased concentration of cryoprotectant also triggers ROCK formation, thus decreases cell survivability.

Cell survivability can be regulated by the rate of nuclei formation in cryoprotectant solution and by the speed of thawing. Nucleation rate “*J*' can be calculated by

(5) $$\begin{array}{c} J=\frac{k\;\Gamma_{z}}{\eta \left(T\right)}\text{exp}\left(\frac{-∆G^{nucl}}{kT}\right), \end{array}$$

where ∆*G*^nucl^ and Γ_z_ are activation energy for nucleation and Zeldovich constant, respectively, and *η* is the viscosity of cryoprotectant solution [49].

It has been observed that a slower rate of thawing increases the temporal hypotonic environment of the cell, but unfortunately, it also enhances cellular toxicity by increasing oxidative stress in the extracellular environment [50]. In order to reduce cellular toxicity, cryoaction can be modified by introducing metallic nanoparticles like Ag and Au. The effect of the mixes of cryoprotector thus obtained leads to formation of amorphous ice. This is because the modified absolute temperature of the vitrified sample goes below glass-transition temperature. The prediction of glass-transition temperature of the newly formed mixture of cryoprotectant solution can be made from mixture property *Y*_mix_. [50]. Here, in our proposed model, *Y*_mix_ is the weighted average of the component properties of *Y*_*i*_ (*i* = cryoprotectant, Ag/Au nanocomposites).

(6) $$\begin{array}{c} Y_{mix}=\frac{\sum Y_{i}f_{i}}{\sum f_{i}}. \end{array}$$

*f*_*i*_ is the fraction of *i*th component within the mixture. Accuracy of mixture property can be enhanced by implementation of Couchman-Karasz and Gordon-Taylor models.

In addition, the nucleation rate in cryoprotectants can be enhanced or modulated by applying a pulsed transverse electric field (E) across the cryoprotectant solution [51]. When molecules of cryoprotectants are polarized in an external electrostatic field (E), then there is force moment “M” acting on the molecules given as *M* = *μ*_o_Esin(*θ*), with *μ*_o_ as the electric dipole moment of the cryoprotectant molecules and *θ* as the angle between dipole moment direction and the field direction, and the potential energy of the interaction *U* is *μ*_o_Ecos(*θ*). As per the Boltzmann distribution law [49, 52], the homogenous distribution of cryoprotectant solution can be represented by

(7) $$\begin{array}{c} f\left(U\right)=A\text{exp}\left(-\frac{U}{kT}\right). \end{array}$$

Thus, it has been shown that cell survivability is highly regulated by nucleation rate, which again varies with activation energy, as shown by (2).

Lastly, input of activation energy enhances elastic deformation of cellular membranes [53]. In combination with the electropositive (or electronegative) treatment of the substrate in the presence of Ag/Au nanoparticles, it increases bending elasticity. Also, controlled reduction in ice-crystal formation during slow thawing can be achieved by increasing the surface roughness and porosity of the substrate. Substrate porosity is also enhanced due to the interaction between Ag/Au nanoparticles and the electropositive (or electronegative) coated substrate surface due to the phenomenon of boundary layer theory.

Thus, increased surface porosity in combination with increased cell membrane elasticity interrupts ROCK formation, which in turn reduces the need for ROCK inhibitors during vitrification of cells following thawing, thereby preventing cellular toxicity.

## 8. Conclusion

Various substrates used in tissue engineering constructs and their ideal characteristics have been discussed. The role of substrates (natural polymers, synthetic polymers, and crystalline substances and metals) in the cryopreservation of constructs is important in several cases where cell viability and proliferation were not affected, and the integrity of scaffold was maintained. Despite this, mechanical properties such as elastic limits, diffusivity, permeability, and thermal properties of the scaffolds were altered resulting in reduced physiological activity of the supported cells. Future challenges should improve parameters such as molecular size, shape, weight, surface to volume ratio, concentration, physical stability, solubility, and thermodynamic properties of the substrates used correlating them with cell surface interaction, growth, differentiation, migration, viability, proliferation, and localization. These will enhance the longevity, endurance, performance, and recovery of the cells/tissues from the cryopreserved state enabling effective tissue banking and transplantation. Despite recent advancements in improving the postthawing survival rate of cells, problems still need to be addressed. The most important factor is the enhancement of cell attachment. Although ROCK inhibitors have definitely improved the postthawing survival of cryopreserved cells, additional studies are still needed to enhance the cell adhesion properties during cryopreservation. In addition, the altered shape of the cells in the presence of ROCK inhibitors should be addressed as the cells regained their original shape once the inhibitor was removed. Finally, we must solve the problem of reduced tendency to differentiate after cryopreservation. The present review elucidates the systemic activation of ROCK through caspase enzyme leading to apoptosis. Stress factors induced due to ultra-low temperature help to trigger this reaction. Incorporation of Au/Ag nanomaterials to modify substrate had also been explained which promises to establish optimized criteria for activation of ROCK and its inhibitors.

## Figures and Tables

**Table 1:** Materials commonly used in the construction of scaffolds.

Substrate type	Substrate composition	Reference
Natural polymers	Cellulose Agarose	Diamantoglou et al. [18]
	Alginate	Li et al. [9]

Synthetic polymers	Polyetherimide	Lützow et al. [28]
	Polyimide	Maenosono et al. [54]
	Polylactic acids	Mohammadi et al. [55]

Crystalline substances	Ceramics	Wiegandt et al. [56]
	Calcium phosphates	Zhang et al. [57]
	Bioglass	Jayabalan et al. [58]

Metals	Titanium	Holtorf et al. [59]
	Alumina	Swan et al. [60]

**Table 2:** Effect of substrate on cryopreservation outcome.

Substrate	Reference	Properties
Alginate fibrin beads	Bhakta et al. [7]	(i) Dehydration of beads causes crumpling (ii) Matricellular environment unaltered (iii) Cell proliferation and viability not affected (iv) Highly biocompatible

Chondrite sheets	Maehara et al. [26]	(i) Structural integrity maintained (ii) Microstructure is not affected (iii) Membranous structure important for functionality

Alginate-poly-L-lysine	Murua et al. [30]	(i) High chemical resistance and stability (ii) Efficient therapeutic effect *in vivo* (iii) Aided in neovascularization leading to higher haemocrit levels (iv) Biosafety and long-term functionality of microcapsules

Agarose microencapsulation	Carlos et al. [61]	(i) Young's modulus reduced (ii) Diffusion coefficient varied (iii) Occurrence of cryopreservative damage (iv) Insulin secretion increment with respect to glucose concentration

Hydroxyapatite scaffolds	Altankov et al. [3]	(i) Detrimental cell surface interaction (ii) Cell detachment (iii) Altered thermal properties (iv) Postthaw viability enhanced correspondingly with number density
